# All the microbiology nematodes can teach us

**DOI:** 10.1093/femsec/fiw007

**Published:** 2016-01-10

**Authors:** Silvia Bulgheresi

**Affiliations:** Department of Ecogenomics and Systems Biology, University of Vienna, Althanstrasse 14, 1090 Vienna, Austria

**Keywords:** nematode, bacterium, symbiosis, ecology

## Abstract

Be it their pervasiveness, experimental tractability or their impact on human health and agriculture, nematode–bacterium associations are far-reaching research subjects. Although the omics hype did not spare them and helped reveal mechanisms of communication and exchange between the associated partners, a huge amount of knowledge still awaits to be harvested from their study. Here, I summarize and compare the kind of research that has been already performed on the model nematode *Caenorhabditis elegans* and on symbiotic nematodes, both marine and entomopathogenic ones. The emerging picture highlights how complementing genetic studies with ecological ones (in the case of well-established genetic model systems such as *C. elegans*) and vice versa (in the case of the yet uncultured *Stilbonematinae*) will deepen our understanding of how microbial symbioses evolved and how they impact our environment.

## Abstract (precis)

Nematode–bacterium associations are major research subjects. Complementing genetic studies with ecological ones is necessary to boost our understanding of how microbial symbioses evolved and how they impact the environment.

“The environment is the soul of things”

 from “The Book of Disquiet” by Fernando Pessoa

## INTRODUCTION

With the turn of the millennium, the application of high-throughput sequencing techniques to environmental microbiology revealed not only that every functional biological system is literally bathing in microbes, but also that these and the macrobes (as science philosopher John Dupre graphically refers to multicellular organisms in his *Processes of Life: Essays in the Philosophy of Biology.* Oxford University Press, 2012). are massively interconnected. Therefore, we can no longer proclaim animals and plants as autonomous entities. Instead, we must regard them as ‘holobionts’, that is, as biomolecular networks including their associated microbes (Rosenberg *et al*. 2007; Bordenstein and Theis 2015). A postmodern synthesis of evolutionary biology (Koonin 2009; Jablonka and Lamb 2014) must therefore take into account that natural selection acts on polygenomic entities (‘hologenomes’), that these are epigenetically connected (Liang *et al*. 2013; Asgari 2014; Knip, Constantin and Thordal-Christensen 2014) and that most genomic entities are likely not transmitted vertically but horizontally (i.e. environmentally; Bright and Bulgheresi 2010).

**Figure 1.**
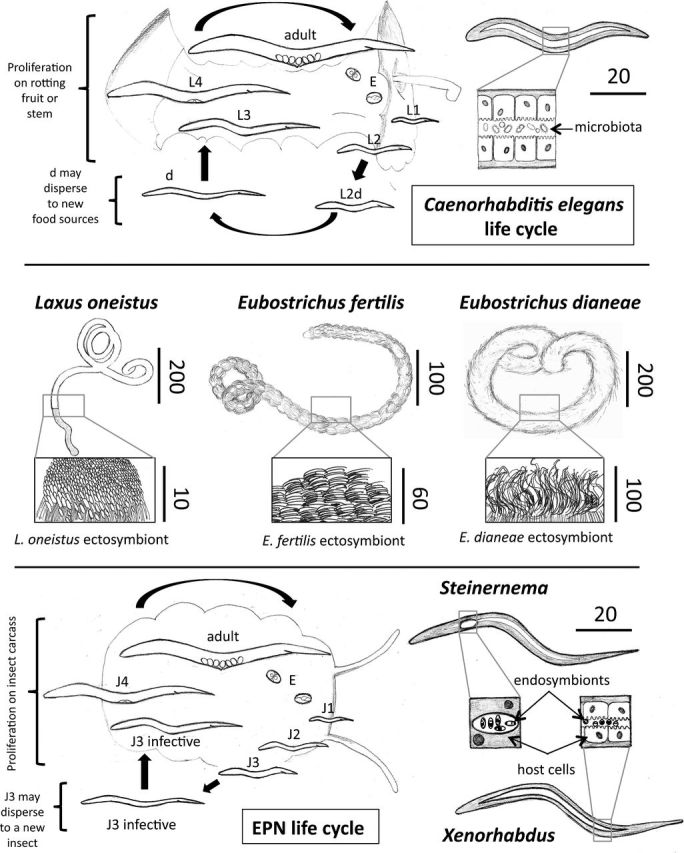
Life cycles of *C. elegans* (top) and of an entomopathogenic nematode (bottom), and nematode symbiont localization in *C. elegans* (top right), three stilbonematid nematodes (center), and two entomopathogenic nematodes belonging to the genera Steinernema and Xenorhabdus (bottom right). L1-L4: larval stages 1–4; d: dauer; J1-4: juvenile stages 1–4; E: embryo; EPN: entomopathogenic nematode. Nematodes and bacterial symbionts sizes are approximate and given in μm. Sketches by Silvia Bulgheresi and Aldo Giannotti.

Nematodes exist in marine, freshwater and terrestrial ecosystems, as well as in plants and animals and may significantly modify them. The experimental tractability or, alternatively, their applied importance have promoted their use in several research areas, including symbiology, immunology and ecology. This is also because many mechanisms underlying stable nematode–bacterium associations are conserved and may therefore provide insights into other systems that affect human well-being such as the gut microbiota. The most-studied nematode, *Caenorhabditis elegans*, is a terrestrial nematode whose relationships with bacteria are predatory (Brenner 1974), defensive (Tan and Shapira 2011) and symbiotic (Portal-Celhay and Blaser 2012; Cabreiro and Gems 2013). Its long experimental history and genetic tractability have made *C. elegans* a very convenient workhorse to investigate numerous biological processes (Blaxter 2011; Xu and Kim 2011), including bacterial pathogenesis and host immunity (Irazoqui, Urbach and Ausubel 2010; Tan and Shapira 2011; Pukkila-Worley and Ausubel 2012). This body of work also has facilitated the advancement of studies of hardly (or not at all) cultivable and genetically non-tractable nematodes such as marine ones coated with thiotrophic bacteria (*Stilbonematinae*), terrestrial insect-killing nematodes associated with *Xenorhabdus* and *Photorhabdus* bacteria, and parasitic filarial nematodes colonized by intracellular *Wolbachia* symbionts (Bulgheresi 2011; Murfin *et al*. 2012; Dillman *et al*. 2012; Slatko *et al*. 2014; see Fig. 1 for drawings of the three nematodes groups discussed here).

## *C. ELEGANS*: A WEALTH OF GENETICS BUT HARDLY ANY ECOLOGY



As poetically phrased in the epigraph to this perspective, and as Petersen and colleagues have reiterated in their timely review (2015) ‘any in-depth understanding of biology requires consideration of the relevant natural context’. Suspiciously, the lack of knowledge about the abiotic and biotic factors free-living *C. elegans* must cope with parallels our unawareness of the function of most of its genes, with almost 70% still lacking validated functional annotation. This is ironic given that *C. elegans* was the first multicellular organism to have its complete genome sequenced (The *C. elegans* Sequencing Consortium 1998). Moreover, both its genome sequence and structure have been subsequently confirmed (e.g. Gerstein *et al*. 2010; Lamm *et al*. 2011; Vergara *et al*. 2014), and the model nematode subjected to many genetic screens (e.g. Lejeune *et al*. 2012; Roy *et al*. 2014). Although less staggering in other model organisms (e.g. *Drosophila*), this situation suggests that only by studying living things in their habitats and as holobionts we will understand the function of many orphan genes in their life cycle and evolution (Rosenberg *et al*. 2007; Bordenstein and Theis 2015). More or less diverse microbial communities likely mediated the origin of multicellular organisms and accompanied them throughout their evolution until present (Alegado *et al*. 2012; Brock *et al*. 2013; Bordenstein and Theis 2015). These microbiota can remain stable after years of laboratory cultivation, influence nutrient metabolism, confer pathogen resistance and affect development (McFall-Ngai *et al*. 2013). In the wild, *C. elegans* inhabits rotting plants, and carries significant amounts of undigested microbes in its gut (Félix and Braendle 2010; Félix and Duveau 2012). However, their potential importance is largely ignored, as inside the lab worms are reared on lawns of the uracil auxotroph *Escherichia coli* mutant strain OP50, and sterilized by sodium hypochlorite (Stiernagle 2006). If we are starting to acknowledge the importance of considering *C. elegans*’ interactions with non-pathogenic, naturally occurring microbes, it is mostly its reaction to standard, cultivable microbes that has been analyzed so far. Although some of these are medically relevant for being human pathogens, we still do not know if *C. elegans* can encounter them in the wild (Cabreiro and Gems 2013; Clark and Hodgkin 2014). Only rare studies have ventured beyond using the model worm gnotobiotically and addressed the role of naturally associated microbes, revealing distinct types of interactions, from deleterious, pathogenic ones involving bacteria, fungi and a virus (e.g. Troemel *et al*. 2008; Hodgkin *et al*. 2013), to beneficial, immunoprotective ones such as those with *Bacillus megaterium* and *Pseudomonas mendocina* (Montalvo-Katz *et al*. 2013). Despite these sparse data, systematic analysis of the *C. elegans* microbiota is still lacking: How stable is it in a worm lifetime and how much is conserved across different worm populations? Which functional gene categories are expressed by the microbiota and how have these influenced major life history characteristics, and what effect have they had on host evolution? Which worm genes are required for establishing and maintaining a healthy microbiota? In short, if model taxa were originally chosen for their undisputable advantages as laboratory systems, we now urgently need to complement this reductionist approach by performing ecological studies (Félix and Braendle 2010; Petersen, Dirksen and Schulenburg 2015).

## STILBONEMATIDS: SOME ECOLOGY BUT NO GENETICS

Almost the opposite is true for stilbonematid nematodes, which can be regarded as ‘naturally gnotobiotic’ systems for which cultivation and genetic tools need to be developed fastly. In stark contrast to *C. elegans*, live specimen of these far less handy nematodes has been studied exclusively at their collection site, mainly at the Caribbean marine station of Carrie Bow Cay, Belize. *Stilbonematinae* do not only thrive in tropical shallow-water sand, but here their accessibility is the highest as they abound throughout the year. Astonishingly, each individual of a given worm species is naturally coated by one phylotype of thiotrophic *Gammaproteobacteria* in a one-to-one (binary) relationship (Ott, Bright and Bulgheresi 2004a,b). As the thiotrophic bacteria are ‘simply’ stuck to the host surface, these associations are referred to as *ecto*symbioses. Notably, *Stilbonematinae* are the only macrobes known to invariably establish binary *ecto*symbioses. Beside this distinguishing quality, the fact that the partners can be easily separated from one another makes *Stilbonematinae* an excellent system for dissecting the molecular base of symbiosis-specificity. Indeed, both host-secreted and microbe-associated molecular patterns can be recombinantly expressed or chemically synthesized and their role in partners’ attachment directly tested (as for the lectins discussed below). Studying how the high level of host-symbiont specificity evolved in a ‘naturally gnotobiotic’ nematode can confirm or complement the knowledge gained from *C. elegans*. This, too, as already said, naturally bears a multispecies microbiota. However, if the individual contribution of a given gut resident is to be dissected out, the model host must be reared germ free or experimentally sterilized and reinfected with one bacterial strain of choice. But there is one additional distinctive quality of marine nematode–bacterium associations: strikingly, also the symbiont spatial disposition on the host surface is exact and faithfully transmitted from one generation to the next (Ott, Bright and Bulgheresi 2004a,b).

### Bacterial epithelia, ropes and furs

How are the stilbonematid symbionts arranged on their host surface? *Laxus oneistus* and *Robbea hypermnestra* nematodes are covered by a single layer of rod-shaped bacteria tightly packed with one another and standing perpendicularly to the worm's surface as to form a columnar epithelium. The *L. oneistus* symbiont is the first bacterium ever shown to divide longitudinally by default, but it is unclear if it is the host who triggers this anomalous division mode (Leisch *et al*. 2012). The filamentous ectosymbionts of *Eubostrichus fertilis* and *E. dianeae*, instead, are attached to the worm cuticle with two or one pole(s), respectively (Polz *et al*. 1992; Pende *et al*. 2014). The first divides by symmetric transverse fission at virtually any length between 4 and 45 μm and forms a bacterial coat resembling a braided rope. The second one is the longest (up to 120 μm) bacterium known to divide by transverse fission and forms a bacterial coat resembling a fur. We currently do not know why the symbionts divide unconventionally or what is the function of a given symbiont arrangement, assuming it is an adaptive trait. As for the host side, a distinctive character unifying all *Stilbonematinae* is a system of unique epidermal organs called glandular sense organs (GSOs) (Bauer-Nebelsick *et al*. 1995). In at least two stilbonematids, GSOs likely mediate symbiosis establishment and maintenance as they secrete the Mermaids, a family of Ca2+-dependent lectins that mediates ectosymbiont aggregation and attachment to the cuticle (Bulgheresi *et al*. 2006, 2011). Although all omics-subjected nematodes were found to express C-type lectins (Murfin *et al*. 2012) and although *C. elegans* turns on or upregulates its corresponding genes in response to microbial infections (Schulenburg *et al*. 2008; Bogaerts *et al*. 2010; Miltsch, Seeberger and Lepenies 2014; Kamaladevi and Balamurugan 2015), the stilbonematid Mermaids were the first and until very recently (Miltsch, Seeberger and Lepenies 2014), the only C-type lectins shown to bind bacteria. But marine nematode lectins do not only provide a molecular basis to symbiosis specificity. They also testified how the study of naturally occurring nematode–bacterium associations can help solving societal problems: recombinant Mermaid was indeed shown to block—among other pathogens (Zhang *et al*. 2006, 2008; Mittal *et al*. 2009; Yang *et al*. 2015)—HIV-1 virus infection of human cells (Nabatov *et al*. 2008).

### Why dressing up?

If the exquisite selectivity of the stilbonematid immune system can clearly teach us a lot, what do we know about the partners’ mutual benefits and about the environmental factors that favored the evolution of stilbonematid symbioses? Ecological studies performed in the 90s suggest that stilbonematids trophically depend on their ectosymbionts, and these, in turn, profit from nematode migrations through the sulfide gradient in the marine sediment (Ott *et al*. 1991). All the molecularly identified ectosymbionts belong indeed to the marine oligochaete and nematode thiotrophic symbiont (MONTS) cluster, which comprises 16S rRNA-gene sequences retrieved from gammaproteobacterial sulfur oxidizers associated with these invertebrates, as well as sequences of environmental origin (Polz *et al*. 1994; Bayer *et al*. 2009; Bulgheresi *et al*. 2011; Heindl *et al*. 2011; Pende *et al*. 2014). The closest cultivable relatives of MONTS members are free-living purple sulfur bacteria (*Chromatiaceae*). Beside the 16S rRNA-gene-based phylogenetic placement, the autotrophy of the symbionts is supported by uptake of 14C bicarbonate (Schiemer, Novak and Ott 1990) and by the presence of RuBisCo enzymatic activity (Polz *et al*. 1992). As for the symbiont sulfur-oxidation capability, it is supported by the ATP sulfurylase and sulfite oxidase enzymatic activities, by the presence of elemental sulfur in symbiotic but not in aposymbiotic *L. oneistus* (Polz *et al*. 1992), and by the cloning of the symbiont aprA gene, encoding the alpha subunit of adenosine-5-phosphosulfate reductase (Bayer *et al*. 2009). Moreover, metabolic studies suggest respiratory reduction of nitrate and nitrite (Hentschel *et al*. 1999). Although recently gained genomic data support all the aforementioned metabolic pathways, in addition to ammonia assimilation, it is unclear how symbiont-synthesized organic compounds are transferred to the host or how the host and symbiont N metabolisms are intertwined (Murfin *et al*. 2012). Besides nutrition, several observations point to an additional role of the bacteria in detoxifying their host's environment: at high sulfide concentrations, *Stilbonematinae* may indeed better tolerate heat than non-symbiotic nematodes (Ott 1995). Moreover, Hentschel *et al*. (1999) showed that freshly collected stilbonematids have much lower internal sulfide and thiosulfate concentrations than cooccurring non-symbiotic nematodes, indicating that thiotrophic symbiont coats may provide an efficient barrier against sulfide poisoning. Despite the already performed ecological studies, we still need to determine the exact physical-chemical parameters and microbial communities characterizing the habitats of the different stilbonematid species (e.g. back-reef versus mangrove shallow-water sediment). Especially in view of the fact that no thiotroph symbiont has been enriched or isolated in the laboratory so far, this information could spur symbiont cultivation.

Despite the fact that *Stilbonematinae* have only been observed alive right upon sampling (or fixed, upon sampling and storage), numerous key questions still tantalize us: why do different host species—even within the same microhabitat—coexist each one carrying its own specific bacterial phylotype? Why are different bacterial phylotypes morphologically different and why are they arranged in different, host-specific spatial dispositions, which are transmitted from generation to generation? What is the ecological and evolutionary significance of a specific ‘symbiosis outfit’? Does it favor vertical transmission of those symbionts that cannot survive free living? Nematodes carrying filamentous symbionts are more abundant in sulfide-rich mangrove sediments than in sulfide-poor back-reef sediments (J.A.O., pers. comm.): Is *Eubostrichus* symbiont filamentation necessary to allow its host to tolerate a sulfide-rich environment? I hope that by applying comparative transcriptomics, proteomics and metabolomics between different species, or between nematodes thriving in different habitats, or carrying different kinds of symbiont coats, we will elucidate whether each symbiont spatial disposition serves a specific, host-symbiont metabolic network, evolved, in turn, as adaptation to a given habitat. Following the identification of molecules or molecular pathways, inactivation of host candidate genes to confirm their function might be achieved using the CRISPR/Cas9 system. This was successfully employed in *Pristionchus pacificus* and might therefore become a powerful tool to determine gene function in non-*Caenorhabditis* nematodes (Witte *et al*. 2015). Further, if some symbiont genomic data and host transcriptomic data are already available (Murfin *et al*. 2012), the genome sequence of at least the best studied stilbonematid, *L. oneistus*, would greatly ease the interpretation of high-throughput data. Filarial parasitic nematodes are as refractory to lab practice as stilbonematids but complete genome sequences of both *Brugia malayi*, which causes lymphatic filariasis, and its *Wolbachia* endosymbiont are already available (Foster *et al*. 2005; Ghedin *et al*. 2007) and have facilitated microarray, transcriptomic and proteomic studies that pinpointed fundamental aspects of these pathogenic nematode–bacterium associations (Murfin *et al*. 2012; Slatko *et al*. 2014).

## THE PERFECT MIDDLE

Entomopathogenic nematodes (EPNs) occupy the perfect middle between *C. elegans* and stilbonematids: they are experimentally tractable but, at the same time, much is known about how they shape the populations of plants and host insects (Campos-Herrera *et al*. 2012; Murfin *et al*. 2012; Hussa and Goodrich-Blair 2013). At least two genera of nematodes, *Steinernema* and *Heterorhabditis*, have evolved symbiotic associations with *Gammaproteobacteria*, *Xenorhabdus* and *Photorhabdus* respectively that enable them to kill insects and feed on their carcasses (Dillman and Sternberg 2012; Dillman *et al*. 2012). A specialized infective stage of EPNs vectors the symbionts within the intestine and releases them upon invasion of an insect host. There, the bacteria mediate insect killing and digestion, and protect the carcass from opportunists. Once the insect resources are consumed, the EPN offspring develop into the colonized infective stage and emerge to hunt for a new insect host (Herbert and Goodrich-Blair 2007; Clarke 2008). Luckily, in both types of associations, bacteria and nematodes can be cultivated independently or together, and molecular genetic techniques are available for the bacteria and, in some cases, for the nematodes (Ciche and Sternberg 2007; Goodrich-Blair 2007; Clarke 2008). This technical tractability has enabled the use of EPNs and bacteria as models of mutualism, virulence, evolution, behavior and ecology (Clarke 2008; Ram *et al*. 2008; Adhikari *et al*. 2009; Bode 2009; Richards and Goodrich-Blair 2009; Eleftherianos *et al*. 2010; Hallem *et al*. 2011; Bashey *et al*. 2012). Furthermore, since these nematode–bacterium complexes are pathogenic toward a wide range of insects, an additional goal in studying EPNs is improving their employability in insect pest control (Stock 2005). In particular, investigators are focusing on identifying EPNs traits associated with insect host range and successful parasitism to help improve their field efficacy, and on identifying EPN symbiont products with insecticidal properties, efforts facilitated by sequencing of both bacterial and nematode genomes (Duchaud *et al*. 2003; Ciche 2007; Wilkinson *et al*. 2009; Chaston *et al*. 2011; Dillman, Mortazavi and Sternberg 2012; Bai *et al*. 2013).

## CONCLUSIONS

In closing, I hope that the marriage of ecological knowledge and experimental tractability will soon be stipulated and contracted in as many nematode–bacterium systems as possible. Their study is of fundamental importance to test existing symbiosis theory (Douglas 2008), including how symbionts enable their hosts to conquer oligotrophic environmental niches, how symbionts evade or tolerate host immunity, how they are transmitted between generations and how symbiosis impacts the evolution of an organism. On one hand, it is important to look at model worms in their natural environmental and microbial milieu and, on the other hand, to develop the necessary tools [e.g. cultivation techniques, genetic platforms (Kumar *et al*. 2012) and manipulation] for mechanistic explorations of less-user friendly ones. Comparison among different systems is critical to nail conserved, fundamental molecular mechanisms.
